# Massive cerebral air embolism following percutaneous transhepatic biliary drainage: A case report

**DOI:** 10.1097/MD.0000000000028389

**Published:** 2021-12-30

**Authors:** Jae Ho Lee, Ha Young Lee, Myung Kwan Lim, Young Hye Kang

**Affiliations:** Department of Radiology, University of Inha College of Medicine, 27 Inhang-ro, Jung-gu, Incheon, Korea.

**Keywords:** air embolism, case report, cerebral air embolism, percutaneous transhepatic biliary drainage

## Abstract

**Rationale:**

Cerebral air embolism from portal venous gas rarely occurs due to invasive procedures (e.g., endoscopic procedures, liver biopsy, or percutaneous transhepatic biliary drainage) that disrupt the gastrointestinal or hepatobiliary structures. Here, we report a rare case of fatal cerebral air embolism following a series of percutaneous transhepatic biliary drainage tube insertions.

**Patient Concerns:**

A 50-year-old woman with a history of cholecystectomy, liver wedge resection, and hepaticojejunostomy for gallbladder cancer presented with altered mental status 1 week after percutaneous transhepatic biliary drainage tube placement.

**Diagnoses:**

Extensive cerebral air embolism and acute cerebral infarction.

**Interventions:**

Brain computed tomography and magnetic resonance imaging, hyperbaric oxygen therapy, medical therapy.

**Outcomes:**

Despite the use of hyperbaric oxygen therapy and medical treatment including vasopressors, the patient eventually died due to massive systemic air embolism.

**Lessons:**

To date, there have been no reports of cerebral air embolism due to percutaneous transhepatic biliary drainage with pronounced radiologic images. We reviewed previously reported fatal cases associated with endoscopic hepatobiliary procedures and assessed the possible mechanisms and potential causes of air embolism.

## 1 Introduction

Cerebral air embolism through the biliary tree is a rare complication usually reported in patients who underwent endoscopic retrograde cholangiopancreatography (ERCP).^[1]^ When air enters the venous system, it can reach the cerebral circulation through cardiac shunts, retrograde cerebral venous embolism, or arteriovenous malformations.^[2]^ Common risk factors for air embolism include anatomic anomalies, malignancies, previous biliary interventions or surgeries, gastrointestinal inflammation, and particular interventional techniques.^[3]^ To the best of our knowledge, cerebral air embolism due to percutaneous transhepatic biliary drainage (PTBD) with pronounced brain computed tomography (CT) and magnetic resonance imaging (MRI) findings has not been reported. Here, we describe a rare case of massive cerebral air embolism after a series of PTBD tube placement in a patient with gallbladder cancerm.

## 2 Case description

A 50-year-old woman visited the emergency room of our hospital for altered mental status 1 week after PTBD. She had a history of gallbladder cancer, which required cholecystectomy, liver wedge resection, and hepaticojejunostomy. The final pathologic stage was T2N0M0. Despite 3 courses of postoperative chemotherapy, endoscopic biopsy confirmed local recurrence in the intrahepatic duct 8 months after the operation. Follow-up abdominopelvic CT revealed multiple metastases in the liver with seeding in the right subphrenic and subhepatic spaces.

Due to the presence of strictures in the anastomotic site and biliary duct caused by the local tumor recurrence and multiple hepatic metastases, PTBD was performed 3 times with biliary tract dilatation and stent placement. However, she presented with fever and pain at the PTBD insertion site with drainage of dark blood in the tube after 5 days.

**Figure 1:**
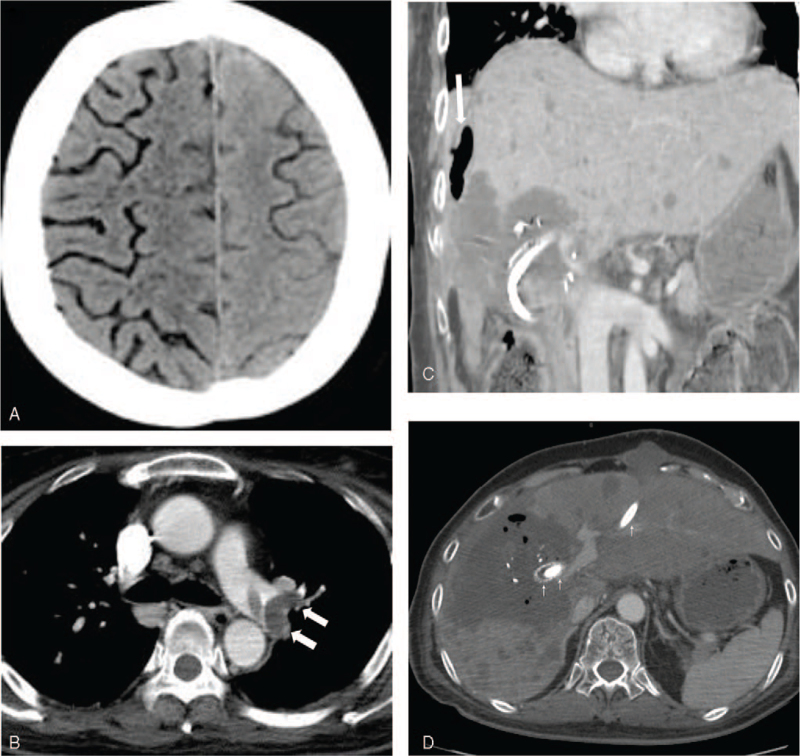
Massive cerebral air embolism after a series of PTBD tube placement in a 50-year-old woman with recurrent gallbladder cancer. Initial noncontrast brain CT shows extensive pneumocephali in the sulci of the right cerebral hemisphere with air densities in the superior frontal sulcus of the left frontal lobe. (B) Pulmonary embolism CT angiography reveals large filling defects in the left main and left lobar (upper and lower) pulmonary arteries arrows, consistent with pulmonary thromboembolism. (C) Coronal and (D) axial abdominopelvic CT shows a ruptured hepatic mass with free air and fluid in the perihepatic space (arrow) and air bubbles around the biliary stent (arrows) and PTBD tube (arrows).

Neurologic examination revealed a semi-comatose state with muscle weakness (muscle strength grade I) in all extremities. Brain CT angiography revealed extensive pneumocephali in the sulci of the right cerebral hemisphere with air densities in the superior frontal sulcus of the left frontal lobe (Fig. 1A). No significant steno-occlusive lesions or filling defects were observed on the scanned neck and intracranial arteries. CT pulmonary angiography showed a large pulmonary thromboembolism in the left main pulmonary trunk and branches of the left pulmonary arteries (Fig. 1B). The patient underwent central venous catheter placement in the right internal jugular vein and hyperbaric oxygen therapy. However, follow-up neurologic examination showed no improvement.

Abdominopelvic CT revealed a large necrotic mass containing air bubbles in the right hepatic lobe, and free air and fluid in the right perihepatic and subphrenic spaces, suggestive of metastatic hepatic tumor rupture (Fig. 1C, 1D). The PTBD tube was inserted into the B3 duct surrounded by a ruptured necrotic hepatic mass (Fig. 1C, 1D). Right portal vein thrombosis and multiple metastatic masses in the subcutaneous and muscle layers of the abdomen were also observed.

**Figure 2:**
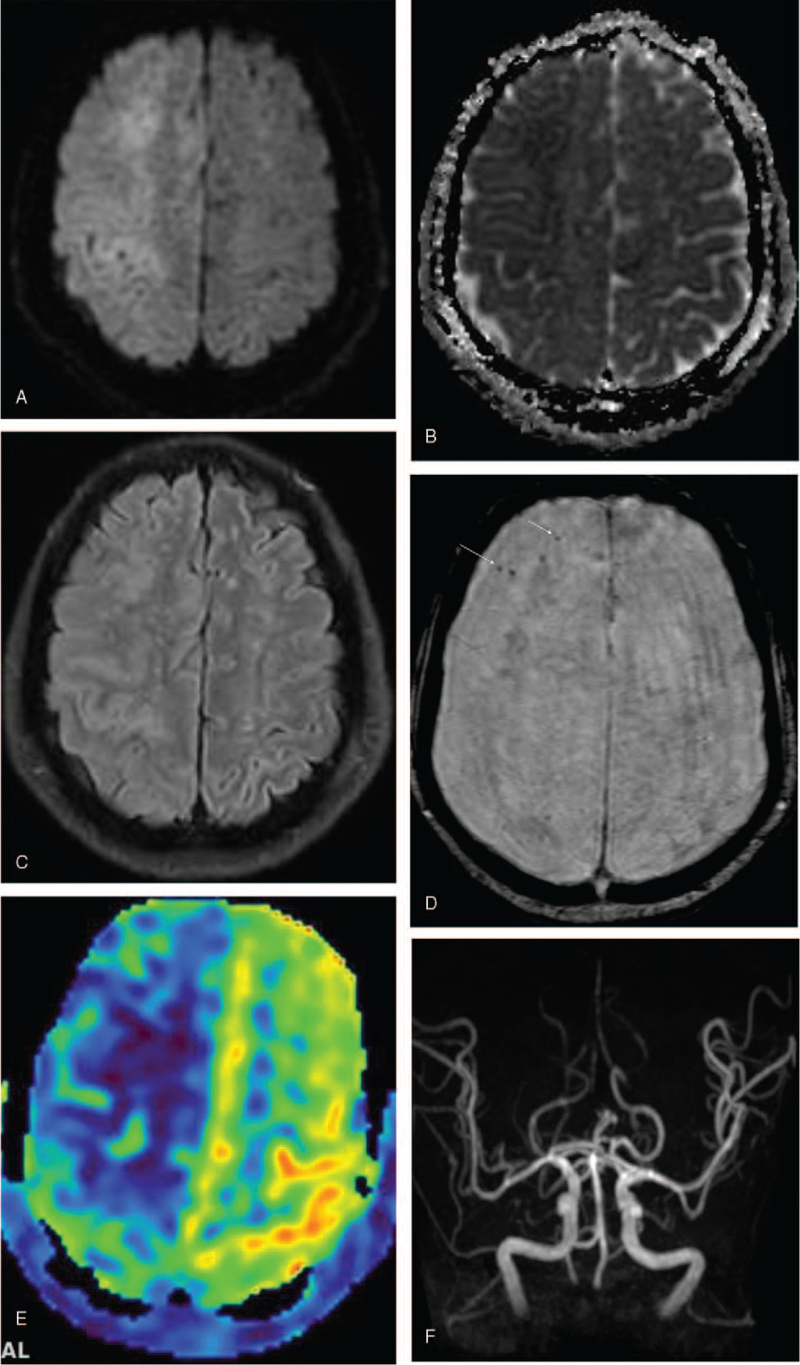
Brain magnetic resonance imaging (MRI) findings. (A) Diffusion-weighted MRI reveals multiple high signal intensities (SI) in the right frontal, right parietal, and left frontal (i.e., superior frontal gyrus) lobes. (B) Apparent diffusion coefficient map shows the corresponding low SI areas. (C) Fluid-attenuated inversion recovery reveals areas of increased SI and cortical swelling, suggestive of acute infarctions. (D) Gradient-echo sequence shows multifocal hypointense blooming dots (arrows) in the right frontal and temporal lobes, consistent with residual air bubbles. (E) Arterial spin-labeling perfusion imaging reveals that the cerebral blood flow is markedly decreased in the right cerebral hemisphere and mildly decreased in the left frontal lobe. (F) Magnetic resonance angiography shows no demonstrable steno-occlusive lesions.

Diffusion-weighted brain MRI taken after 6 hours revealed multiple high signal intensities (SI) in the right frontal, right parietal, and left frontal (i.e., superior and medial gyri) lobes, mainly in the cortex, with low SI on the corresponding apparent diffusion coefficient map (Fig. 2A, 2B). Fluid-attenuated inversion recovery showed increased SI with cortical swelling (Fig. 2C). Gradient-echo sequence revealed multifocal hypointense blooming dots in the right frontal and temporal lobes, suggestive of residual air bubbles (Fig. 2D). Arterial spin-labeling perfusion imaging showed decreased blood flow in the right cerebral hemisphere and left frontal lobe (Fig. 2E). These MRI findings were suggestive of acute infarction in the right cerebral hemisphere and left superior frontal lobe, probably due to cerebral air embolism. Magnetic resonance angiography showed no significant vascular abnormalities (Fig. 2F).

**Figure 3:**
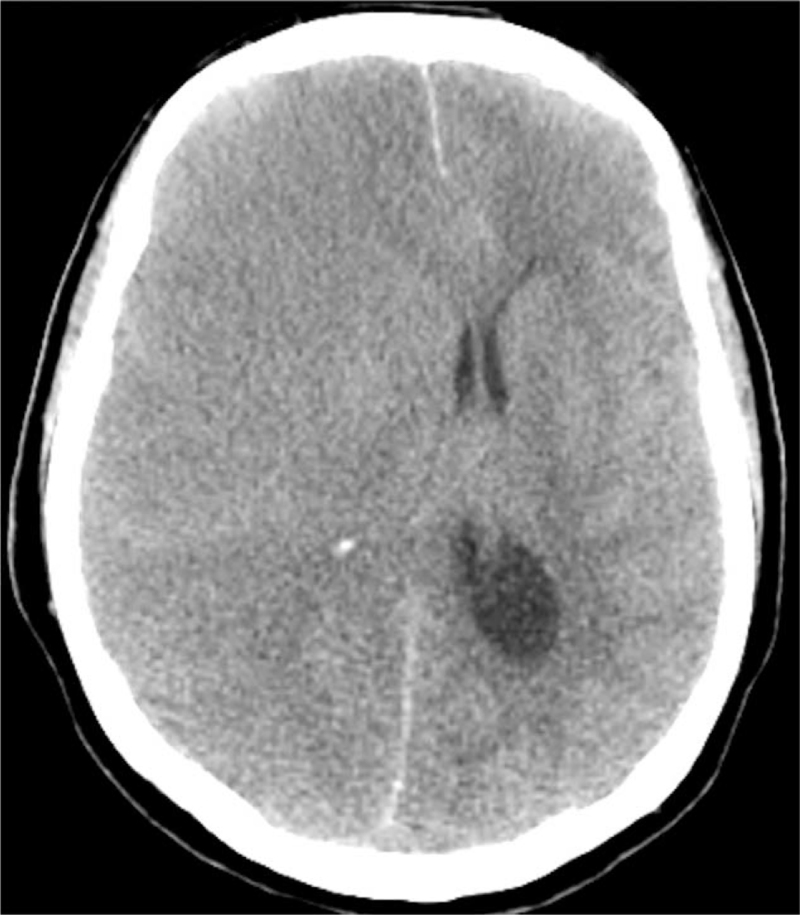
Follow-up brain computed tomography taken after 2 days shows a marked increase in the extent of infarct-related edema in the right cerebral hemisphere with left-sided midline shifting.

Follow-up brain CT taken 12 hours later showed expansion of the infarct-related edema in the right cerebral hemisphere with left-sided midline shifting (Fig. 3). Asymmetric enlargement of the left lateral ventricle was also observed, consistent with obstructive hydrocephalus. Despite the use of vasopressors, the patient's blood pressure continued to drop. She began to develop progressive oliguria, metabolic acidosis, and hyperkalemia. The patient died 12 days after admission.

## 3 Discussion

Systemic air embolism is a rare complication of esophagogastroduodenoscopy and biliary procedures, including ERCP and PTBD.[^4^,^5^] To the best of our knowledge, systemic or cerebral air embolism in association with PTBD alone has not been reported. We have described a rare case of acute diffuse cerebral infarction due to massive air embolism in a 50-year-old woman with recurrent gallbladder cancer following PTBD. We hypothesized that the air in the biliary tree entered the venous system through the portal/hepatic veins or biliary-venous fistula, which resulted in fatal cerebral air embolism.

Cerebral air embolism is an extremely rare and potentially fatal complication that can occur during or after ERCP. The majority of reported cases of cerebral air embolism were fatal because these may present with immediate cardiopulmonary collapse.[^3^,^6^] Among 11 cases of cerebral embolism during or after ERCP and 10 cases during or after esophagogastroduodenoscopy, Park et al^[7]^ reported 3 fatal cases each associated with ERCP and invasive procedures (e.g., liver biopsy and esophageal dilatation). Some patients either had a shunt (n = 12) or a patent foramen ovale (PFO) (n = 7).^[7]^ In a cohort of patients who underwent ERCP, Finsterer et al^[6]^ reported that while 9 of 14 patients (64%) with systemic air embolism died, only 1 of 6 patients (17%) with regional air embolism had a fatal outcome. Hence, the amount and extent of air embolism are potential prognostic factors. Shaikh and Ummunisa^[8]^ reported that vascular air embolism has a mortality rate of 48% to 80%.

Maccarone et al^[4]^ described 2 cases of cerebral air embolism after PTBD and subsequent ERCP. The first patient had pulmonary outflow obstruction and PFO on transesophageal echocardiography and presented with bleeding on the insertion site. In both patients, blood clots were obtained from the ampulla during ERCP. These findings suggest the importance of considering the possibility of air embolism in cases of blood clots following ERCP, PTBD, or other hepatic interventions.[^4^,^6^,^9^,^10^,^11^] Although the authors did not explain the exact etiology of the blood clot, venous injury or bilio-venous shunt formation during the PTBD are potential causes. In our case, the patient had extensive hepatic necrosis and blood drainage through the PTBD tube. This suggests that tumor rupture could have triggered air embolism through the tube.

Various mechanisms for air embolism due to ERCP have been suggested, including mechanical irritation of the bile duct wall (by the endoscope or bile duct stones), development of bilio-venous shunts, spontaneous transgression of air from bile ducts,^[12]^ irritation of the bile duct walls by contrast injection with subsequent air leakage into the venous system, clearing of metallic bile duct stents, and air migration to the portal vein via sphincterotomy.[^10^,^11^] In our patient, consecutive PTBD tube insertions are thought to have disrupted the liver architecture, creating fistulas between the biliary and venous system. This could provide direct communication to facilitate air embolism. Furthermore, she had other risk factors, including underlying malignancy and metal biliary stent placement. Rupture of the metastatic hepatic mass could also facilitate air leakage through the drainage site, resulting in massive venous air embolism. Additionally, large pulmonary embolism associated with the underlying malignancy is suspected to be responsible for the cardiovascular collapse and transpulmonary venous-to-arterial air passage, resulting in paradoxical cerebral air embolism. However, the presence of a PFO was not evaluated in our patient. Nevertheless, air embolism should be considered as a differential diagnosis in patients who underwent ERCP and other high-risk hepatobiliary procedures presenting with cardiopulmonary compromise.^[13]^

We have reported a rare case of massive cerebral air embolism after a series of PTBD in a patient with recurrent gallbladder cancer. Liver architecture disruption via multiple PTBD tube insertions, hepatic mass rupture, and concurrent large pulmonary embolism are thought to be the potential causes of massive air embolism in our case. Early diagnosis and timely management are crucial in improving the clinical outcomes of this potentially fatal condition.

## Author contributions

**Conceptualization:** Jae Ho Lee, Ha Young Lee.

**Investigation:** Jae Ho Lee, Ha Young Lee.

**Methodology:** Jae Ho Lee, Ha Young Lee.

**Writing – original draft:** Jae Ho Lee, Ha Young Lee.

**Writing – review & editing:** Myung Kwan Lim, Young Hye Kang.
